# IMPLEMENTATION BARRIERS AND FACILITATORS OF COGNITIVE STIMULATION THERAPY ACROSS CARE SETTINGS

**DOI:** 10.1093/geroni/igac059.2264

**Published:** 2022-12-20

**Authors:** Milta Little, Kim Johnson, Francis Karia, Eleanor McConnell

**Affiliations:** Duke University, Hillsborough, North Carolina, United States; Duke University, Durham, North Carolina, United States; Duke University, Durham, North Carolina, United States; Duke University, Durham, North Carolina, United States

## Abstract

Cognitive Stimulation Therapy (CST) is an evidence-based treatment for people living with dementia that is not in widespread use in the United States. To better understand barriers and facilitators to implementation of CST, we conducted virtual focus groups of newly trained facilitators of Cognitive Stimulation Therapy (CST). Of 12 facilitators trained, representing two settings of care (The Program for All-inclusive Care of the Elderly and a Continuing Care Retirement Community), 4 facilitators, representing Social Work n = 2, Speech Therapy n = 1, and Recreation Therapy n = 1, participated. We analyzed interview transcriptions using framework analysis guided by constructs from the Consolidated Framework for Implementation Research (CFIR). Themes that emerged across constructs were 1) balancing competing personal, stakeholder, and organizational needs; 2) lack of mastery experiences and the need for opportunities for more practice, reflection, and feedback from trainers; 3) building a community of facilitators to provide peer support; 4) logistical concerns; and 5) identifying appropriate participants who would most benefit from CST. The barriers to implementation imposed by the ongoing COVID-19 pandemic were also a cross-cutting theme. Many of these barriers are readily managed, and professionals who implement CST in their sites should be aware of and take into consideration these barriers and facilitators.

